# Preliminary Clinical Outcomes of Biotherapy Combined With Rehabilitation for the Treatment of Knee Osteoarthritis: A Retrospective Observational Study

**DOI:** 10.7759/cureus.109836

**Published:** 2026-05-28

**Authors:** Tomohiro Oka, Katsuyoshi Tanaka, Kai Nakagawa, Masaaki Ishida, Kei Noguchi

**Affiliations:** 1 Rehabilitation/Physical Therapy, Kobegakuin University, Kobe, JPN; 2 Department of Physical Therapy, Bukkyo University, Kyoto, JPN; 3 Rehabilitation, C-FORTE Inc., Ashikaga City, JPN

**Keywords:** biologic therapies, clinical outcomes, knee osteoarthritis/ koa, musculoskeletal rehabilitation, platelet-rich plasma (prp)

## Abstract

Background: Knee osteoarthritis (KOA) is a common musculoskeletal condition that markedly reduces patients’ quality of life and places an increasing burden on healthcare systems. Biotherapy has anti-inflammatory and tissue-reparative effects but does not adequately address KOA-related biomechanical or functional impairments. In contrast, rehabilitation improves muscle function and gait mechanics. Although combining these approaches may improve outcomes in patients with KOA, related clinical evidence remains limited.

Objective: This study aimed to preliminarily investigate the combined effects of platelet-derived factor concentrate-freeze-dried (PFC-FD) injection therapy and rehabilitation in patients with KOA at one and three months following injection.

Methods: This retrospective observational study analyzed data of 17 patients with KOA who underwent three sessions of freeze-dried platelet-derived factor concentrate injections, followed by at least three sessions of standardized rehabilitation. Patient-reported outcomes were assessed at baseline and one month and three months after treatment, using the Japanese version of the Knee Injury and Osteoarthritis Outcome Score (J-KOOS). The primary outcome was response rate, which was defined according to the Outcome Measures in Rheumatology-Osteoarthritis Research Society International criteria. Longitudinal changes in J-KOOS scores were analyzed using a linear mixed-effects model.

Results: Among the 17 patients assessed (median age, 68 years; 71% women), 9 (52.9%) met the responder criteria at one month and 12 (70.6%) at three months. The response rate and effect sizes for the functional subscales (e.g., quality of life: d = 1.62) were comparatively higher than those typically reported for PFC-FD injection alone.

Conclusion: The combination of PFC-FD injection and rehabilitation showed potentially favorable clinical outcomes, particularly for improving physical function and quality of life. Given the preliminary and non-controlled nature of this study, these findings should be interpreted with caution. Future controlled studies are warranted to confirm whether rehabilitation augments the clinical benefits of injection therapy.

## Introduction

Knee osteoarthritis (KOA) is one of the most prevalent musculoskeletal disorders worldwide and significantly impairs patient quality of life (QOL), posing substantial direct, indirect, and intangible burdens on healthcare systems and society [1-5]. KOA is highly prevalent in Japan, affecting an estimated 25.3 million Japanese individuals aged 40 years and older [6], underscoring its significance as a major public health concern in an increasingly aging society. Standard KOA management typically begins with non-pharmacological interventions, particularly exercise therapy, followed by pharmacological treatments if sufficient symptom relief is not achieved [7-9]. In severe cases where conservative measures fail, surgical options such as total joint replacement may be considered. However, while surgical interventions are invasive and may not be appropriate for patients who wish to avoid such procedures, pharmacological treatments have limited efficacy and may be associated with adverse effects [10]. These limitations highlight the urgent need for alternative therapeutic strategies to address the increasing burden of KOA.

Biotherapies such as platelet-rich plasma (PRP) have gained increasing attention as potential disease-modifying treatments for KOA because they deliver growth factors and cytokines that promote tissue repair and modulate inflammation (collectively referred to as "biotherapy" in this study). Among these, platelet-derived factor concentrate-freeze-dried (PFC-FD), a freeze-dried formulation of platelet-derived growth factors enabling standardized delivery and long-term storage, has been investigated as an alternative to standard PRP [11]. Several systematic reviews have reported pain relief and functional improvement following PRP injections, suggesting their potential as alternative options when conventional conservative treatments are insufficient [12-14]. However, the effects of biotherapy appear to be largely limited to short-term improvements in pain and function, and long-term clinical relevance remains unclear [15].

Although biotherapies target biological processes, including inflammation and tissue regeneration, they may not adequately address functional or psychosocial impairments, such as muscle dysfunction or abnormal movement patterns. In contrast, rehabilitation, including exercise therapy and patient education, aims to enhance muscle performance, optimize movement patterns, and strengthen self-management skills. Therefore, rehabilitation offers a complementary strategy that addresses aspects that are not directly targeted by biotherapy. Hence, combining regenerative therapy with structured rehabilitation may be a promising approach for improving therapeutic outcomes. However, the evidence from studies examining their combined effects remains limited [16]. Thus, this preliminary study aimed to evaluate the effects of combining, specifically, the available evidence comprising only a small number of studies with heterogeneous PRP formulations and exercise protocols has not yet examined the use of PFC-FD in combination with a structured, phased rehabilitation program, nor has it employed internationally validated responder criteria Outcome Measures in Rheumatology Alinical Trials-Osteoarthritis Research Society International (OMERACT-OARSI) to evaluate clinical outcomes [16]. Furthermore, no study to date has investigated this combined approach in a Japanese clinical cohort, where patient demographics and clinical characteristics may differ from those in Western populations. Thus, this preliminary study aimed to evaluate the effects of combining PFC-FD injection therapy and rehabilitation in patients with KOA at one and three months following injection, using international assessment criteria. This study intends to offer preliminary insights to inform the design of future large-scale prospective studies.

## Materials and methods

Study design and subjects

**Figure 1:**
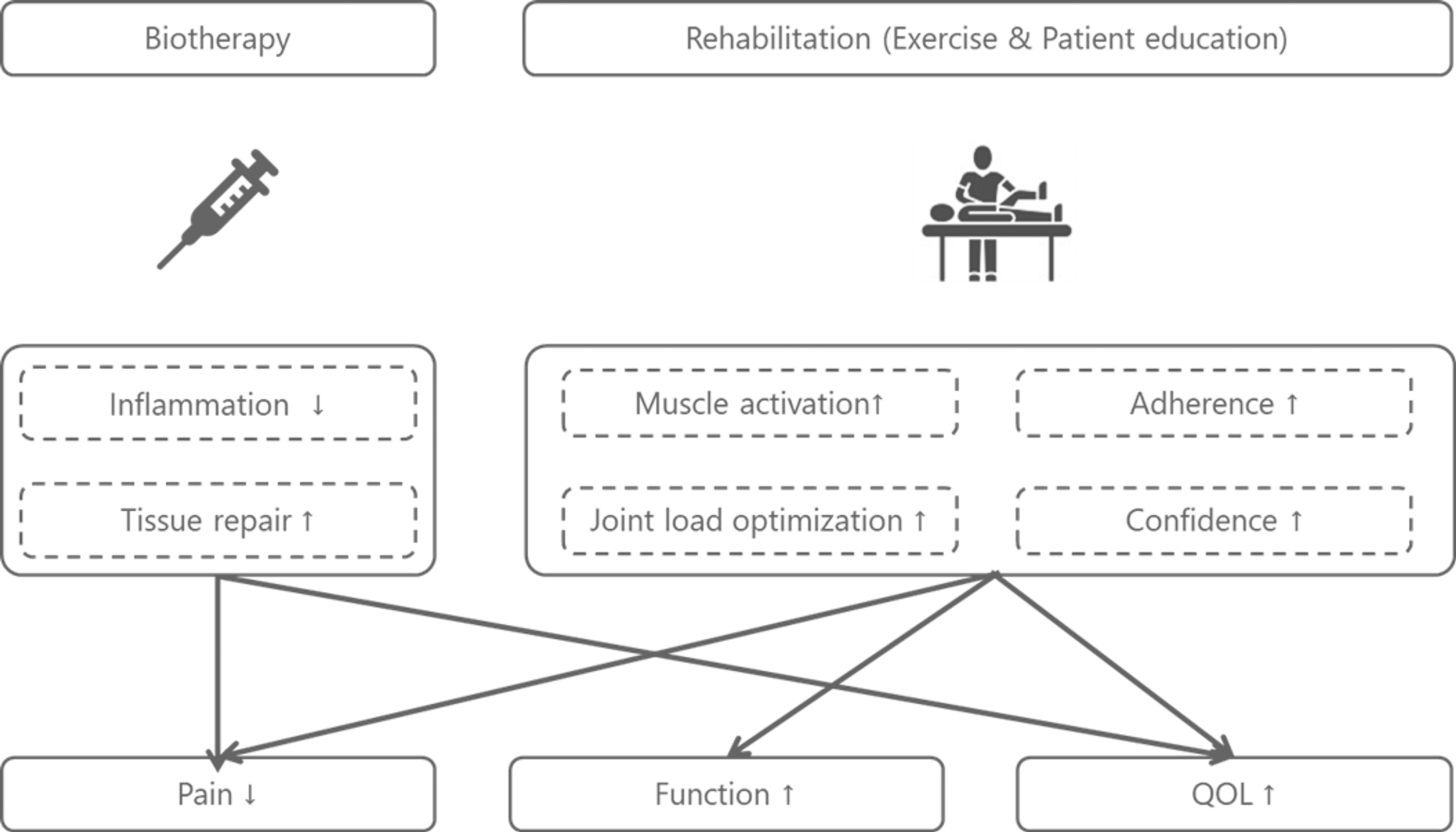
Conceptual framework of integrated biotherapy and rehabilitation for knee osteoarthritis

This study investigated the integrated effects of biotherapy and rehabilitation for knee osteoarthritis, as conceptually illustrated in Figure 1. Both institutions are private, non-insurance-covered medical providers. Inclusion criteria were as follows: (1) adults aged 18 years or older with a clinical and radiographic diagnosis of KOA (Kellgren-Lawrence (KL) grade I-IV); (2) completion of three sessions of PFC-FD injection therapy; and (3) completion of at least three sessions of standardized rehabilitation. Exclusion criteria included a history of lower-limb joint surgery and inability to provide informed consent. Baseline demographic and clinical characteristics, including age, sex, body mass index (BMI), and radiographic severity, assessed using the Kellgren-Lawrence grade, were extracted from patients’ medical records. The authors’ affiliated institutions approved of this study. This study was approved by the Ethics Committee of C-FORTE Inc. (Approval No. 003; Date: September 17, 2025). All participants provided written informed consent in accordance with the Declaration of Helsinki.

Preparation of the injectable formulation

Platelet-derived factor concentrate is derived from PRP [17]. PFC-FD is a freeze-dried formulation developed to stabilize PRP components and enable long-term storage. PFC-FD contains growth factors similar to those found in PRP and has been reported to potentially contribute to the improvement of clinical symptoms in patients with KOA [18]. In this study, three sessions of PFC-FD injection were administered, following the established clinical protocol reported by Ohtsuru et al., in which this regimen was demonstrated to be safe and effective in patients with KOA [18]. For preparation, 40 mL of whole blood was collected from each patient and processed at a central cell-processing laboratory (CellSource, Tokyo, Japan) to produce PFC-FD through a standardized centrifugation and freeze-drying procedure. The resulting solution was filtered through a 0.45-μm membrane for sterilization and subsequently freeze-dried to produce PFC-FD, which was reconstituted in 6 mL of sterile saline before intra-articular injection.

Rehabilitation protocol

Rehabilitation after PFC-FD injection was implemented in five sequential phases depending on the post-injection time point. The objectives and interventions for each phase were as follows. Phase 1 (post-injection week 0-1): Walking activity was restricted to facilitate the absorption of the intra-articularly injected platelet components. The patients received education on self-management of the affected knee and were instructed to perform mild periarticular exercises. Phase 2 (weeks 2-4: Walking restrictions were lifted; however, sports and recreational activities remained prohibited. Non-weight-bearing exercises were prescribed to support pain control and maintain joint range of motion. Phase 3 (weeks 5-8): Exercises aimed at improving gait mechanics were introduced if pain did not worsen or if residual joint effusion was absent. Phase 4 (weeks 9-12): After confirming the absence of recurrent pain or effusion, progressive lower-limb functional training was implemented to enable single-leg stair climbing. Phase 5 (week 13 onward): If no signs of pain or effusion were present, a gradual return to sport-specific activities was initiated through targeted exercises.

Outcomes

Assessments were conducted at three time points: before injection and at one and three months after injection. Patient-reported outcomes were evaluated using the Knee Injury and Osteoarthritis Outcome Score (KOOS), which is widely used internationally to assess patients with KOA [19]. The reliability and validity of its Japanese version (J-KOOS) are well established. J-KOOS consists of the following five subscales: pain, symptoms, activities of daily living (ADL), sport and recreation function, and QOL. Each subscale is scored on a scale of 0 (extreme problems) to 100 (no problems) [20]. The primary outcome was defined according to the Outcome Measures in Rheumatology and Osteoarthritis Research Society International (OMERACT-OARSI) responder criteria [21]. A patient was classified as a responder if either of the following criteria was met: 1) a relative improvement of ≥50% and an absolute improvement of ≥20 points in either the pain or physical function subscale; or 2) a relative improvement of ≥20% and an absolute improvement of ≥10 points in at least two of the following three subscales: pain, physical function, and QOL.

Statistical analysis

The data were summarized using descriptive statistics. Continuous variables are presented as medians with interquartile ranges (IQRs) and categorical variables as frequencies and percentages. The OMERACT-OARSI responder rate was calculated from the KOOS scores. Longitudinal changes in the KOOS total and subscale scores were analyzed using a linear mixed-effects model, with time as a fixed effect and age, sex, BMI, and KL grade as covariates, given prior evidence of their influence on worsening pain and declining function in KOA [22]. All statistical analyses were performed using EZR (version 1.61; Saitama Medical Center, Jichi Medical University, Saitama, Japan) [23], which served as the graphical user interface for R. A two-sided significance level of 5% was considered significant. To determine treatment effect magnitude, effect sizes (Cohen’s d) were calculated for changes in J-KOOS scores. Additionally, to contextualize clinical relevance, the odds ratio (OR) for the responder rate was calculated and compared with historical control data from a previous study using the same biotherapy protocol [18]. No missing data were identified for the primary outcome measure (J-KOOS) at any of the assessed time points. As this was a preliminary retrospective study, a formal a priori sample size calculation was not conducted.

## Results

Overall, 17 participants were included in the study. Their median age and BMI were 68.0 years (IQR, 53.0-82.0) and 23.45 kg/m² (IQR, 20.20-30.10), respectively, and 12 (70.6%) participants were female. According to the KL grade, two (11.8%), four (23.5%), seven (41.2%), and four (23.5%) participants had grade I, II, III, and IV disease, respectively. The participants attended a median of seven rehabilitation sessions (IQR, 6-9) (Table 1).

**Table 1:** Demographic and clinical characteristics of the participants Quantitative variables are presented as median (interquartile range), and categorical variables as number (percentage). KL, Kellgren–Lawrence; BMI, body mass index; IQR, interquartile range

Variables		Overall (n=17)
Age (y)		68.00 (53.00, 82.00)
Height (cm)		160.00 (148.00, 169.00)
Weight (kg)		60.00 (48.00, 82.00)
BMI (kg/m^2^)		23.45 (20.20, 30.10)
Rehabilitation sessions (times)		7 (6, 9)
Female		12 (70.6)
Injection side	Right	8 (47.1)
	Left	7 (41.2)
	Bilateral	2 (11.8)
KL grade	I	2 (11.8)
	II	4 (23.5)
	III	7 (41.2)
	IV	4 (23.5)

According to the OMERACT-OARSI criteria, 9 (52.9%) and 12 (70.6%) of the 17 participants were classified as responders at one and three months after injection, respectively. The response rate in this study was higher than the historical control rate of 54.8% (142/259) reported by Ohtsuru et al. [18], yielding an OR of approximately 1.98, suggesting a clinically meaningful advantage of the combined approach. Table 2 reports the median values and adjusted changes in J-KOOS scores over time.

**Table 2:** Adjusted changes in J-KOOS over time J-KOOS: Japanese version of the knee injury and osteoarthritis outcome score, IQR: interquartile range, CI: confidence interval, ADL: activities of daily living, QOL: quality of life. Adjusted general linear mixed-effects models with time as a fixed effect and subject as a random effect, adjusting for age, sex, BMI, and KL grade. **p <.01. ^1^Pairwise comparison between baseline and one month post-injection. ^2^Pairwise comparison between baseline and three months post-injection.

Outcomes	Baseline (Median (IQR))	Post-Injection 1 month (Median (IQR))	Adjusted Estimate (95% CI)^1^	Post-Injection 3 months (Median (IQR))	Adjusted Estimate (95% CI)^2^
J-KOOS Total	54.17 (51.79, 64.28)	73.22 (62.35, 79.46)	17.75 (11.18–24.32)**	75.59 (66.07, 85.72)	21.61 (15.19–28.05)**
ADL	72.06 (63.24, 79.41)	85.29 (75.74, 88.24)	15.90 (8.95–22.85)**	88.24 (76.47, 98.53)	18.20 (11.39–25.01)**
Pain	58.33 (50.00, 69.44)	77.78 (65.28, 81.25)	20.99 (12.80–29.17)**	80.56 (69.44, 88.89)	24.13 (16.11–32.16)**
QOL	25.00 (18.75, 31.25)	43.75 (29.69, 56.25)	23.73 (13.89–33.57)**	56.25 (43.75, 68.75)	32.81 (23.18–42.44)**
Sports and recreation	30.00 (20.00, 40.00)	45.00 (20.00, 67.50)	20.99 (7.34–34.63)**	50.00 (25.00, 85.00)	27.19 (13.83–40.55)**
Symptom	60.71 (39.29, 75.00)	71.43 (58.92, 83.03)	12.32 (5.84–18.80)**	75.00 (67.86, 78.57)	16.30 (9.96–22.63)**

Significant increases were observed in total J-KOOS score and the scores for all five subscales (pain, symptoms, ADL, sport and recreation, and QOL) at both one and three months after injection compared with baseline. The calculated effect sizes (Cohen’s d) for the changes were large to very large, indicating substantial clinical improvements beyond statistical significance: 1.43 for pain, 0.97 for sport and recreation, and 1.62 for QOL. No serious adverse events were observed during the study period. Minor transient local reactions-including mild swelling at the injection site, transient pain, and warmth-were noted in some participants, consistent with findings previously reported for PFC-FD [18].

## Discussion

This preliminary study investigated the combined effects of PFC-FD injection therapy and rehabilitation in patients with KOA. The response rate increased from 52.9% at one month to 70.6% at three months after injection therapy. The J-KOOS scores also significantly increased over time. These findings suggest that integrating regenerative therapy with structured rehabilitation may potentially yield additive or complementary effects on reducing pain, improving physical function, and enhancing the QOL in patients with KOA, as conceptualized in Figure 1. However, given the absence of a comparator group, causal attribution to either component of the intervention cannot be made from this study alone.

The response rate observed in this study (70.6% at three months) exceeded that of 54.8% (142/259) reported for PFC-FD monotherapy in a previous controlled study [18]. This difference yielded an OR of approximately 1.98, suggesting that the combination of rehabilitation and biotherapy nearly doubled the likelihood of achieving a clinically meaningful response. Furthermore, a comparison of effect sizes revealed the distinct added value of rehabilitation. While a previous study on PFC-FD monotherapy reported a “large” effect size for pain (assessed using a visual analog scale) but only “moderate” effect sizes for KOOS subscales at three months [18], our study demonstrated “large” to “very large” effect sizes (Cohen’s d: >0.9) not only for pain (d = 1.42) but also for sports and recreation (d = 0.97) and QOL (d = 1.62) subscales. This discrepancy highlights a critical insight: while biotherapy primarily targets the biological aspect of the pathology to reduce pain, structured rehabilitation is essential for translating pain relief into improved functional performance and daily activities. In other words, the “moderate” functional outcomes typically seen with biotherapy alone can be elevated to “large” outcomes through the complementary integration of rehabilitation. It should be noted, however, that this interpretation is exploratory; without a rehabilitation-alone arm, the independent contribution of rehabilitation to the observed functional gains cannot be directly quantified.

The J-KOOS scores showed significant improvements over time. However, the timing at which each subscale exceeded the minimal clinically important difference (MCID, 10 points) varied [24]. At one month post-treatment, the pain and QOL subscale scores exceeded the MCID threshold, whereas at three months, the ADL, pain, QOL, and sport and recreation subscale scores surpassed the threshold. The early changes in the pain and QOL scores may reflect the anti-inflammatory effects of the biotherapy in combination with basic functional recovery facilitated by rehabilitation. However, improvements in ADL and sport and recreation scores were observed later; this may be attributed to the gradual progression of weight-bearing and advanced functional exercises in rehabilitation, which require more time to yield measurable gains. In contrast, the lower bound of the 95% CI for the symptom subscale scores did not exceed the MCID. The symptom subscale included swelling, sensation of joint catching, limited range of motion, and stiffness. Although no serious adverse events have been reported in previous studies investigating the effects of PFC-FD, mild local reactions have been observed, such as swelling at the injection site, transient pain, and warmth [18]. These transient reactions may have influenced the symptom scores and could partially explain why the score improvements for this subscale did not exceed the MCID. However, reports regarding the combined use of biotherapy and rehabilitation are limited [16,25]. Biotherapy is generally understood to exert its effects at the molecular, cellular, and tissue levels, whereas rehabilitation targets the organ, individual, and societal levels [26]. Although this was a preliminary investigation, its significance lies in its demonstration of the potential clinical utility of an integrative approach. In addition, the present findings offer insights that may inform the design of future prospective trials. Importantly, identifying the specific functional domains that are improved through rehabilitation is a priority. Future studies should incorporate objective assessments of quadricep strength, gait biomechanics, and other indicators of muscle function and movement patterns.

This study had some limitations. First, and most critically, the absence of a control group (e.g., PFC-FD injection alone or rehabilitation alone) represents a fundamental methodological constraint; without a comparator arm, any interpretation of additive or complementary effects remains speculative. Second, the retrospective design carries inherent risks of information bias and unmeasured confounding. The number of rehabilitation sessions varied among participants, and data on important covariates-such as concomitant NSAID use, home exercise adherence, and other lifestyle variables-were not systematically recorded; future prospective studies should carefully control for these variables. Third, this study relied exclusively on patient-reported outcomes, and no objective functional measures, such as quadriceps strength or gait analysis, were incorporated, limiting the ability to elucidate the mechanisms through which rehabilitation may augment the effects of injection therapy. Fourth, because all participants received out-of-pocket treatments at specialized private clinics, a substantial selection bias cannot be excluded, which may have amplified the observed treatment response and limits the generalizability of the findings. Finally, as this was a preliminary retrospective study with a small sample size and no formal power calculation, the findings should be interpreted as exploratory rather than confirmatory evidence; nevertheless, they provide a basis for future large-scale, controlled prospective trials.

## Conclusions

This retrospective observational study provides preliminary evidence suggesting that the combination of PFC-FD injection therapy and rehabilitation may yield clinically meaningful improvements in pain, physical function, and QOL in patients with KOA. The comparison of effect sizes with historical data raises the hypothesis that rehabilitation may complement the biological effects of injection therapy, particularly by translating pain relief into functional recovery in domains where injection therapy alone may be insufficient. However, given the absence of a control group, the retrospective design, and the small sample size, these findings are exploratory and must be interpreted with caution. As one of the few studies to investigate this combined approach in Japan, the present study underscores the need for large-scale, controlled prospective research to confirm the reproducibility of these findings, clarify the underlying mechanisms, and determine the optimal components and dosage of rehabilitation.
